# Recent Findings in the Posttranslational Modifications of PD-L1

**DOI:** 10.1155/2020/5497015

**Published:** 2020-01-09

**Authors:** Shu-Man Li, Jie Zhou, Yun Wang, Run-Cong Nie, Jie-Wei Chen, Dan Xie

**Affiliations:** ^1^State Key Laboratory of Oncology in South China, Collaborative Innovation Center for Cancer Medicine, Sun Yat-sen University Cancer Center, Guangzhou 510060, China; ^2^Department of Hematologic Oncology, Sun Yat-sen University Cancer Center, Guangzhou 510060, China; ^3^Department of Pathology, Sun Yat-sen University Cancer Center, Guangzhou 510060, China

## Abstract

Immune checkpoint therapy, such as the reactivation of T-cell activity by targeting programmed cell death 1 (PD-1) and its ligand PD-L1 (also called B7-H1 and CD274) has been found pivotal in changing the historically dim prognoses of malignant tumors by causing durable objective responses. However, the response rate of immune checkpoint therapy required huge improvements. It has been shown that the expression of PD-L1 on cancer cells and immune cell membranes is correlated with a more durable objective response rate to PD-L1 antibodies, which highlights the importance of deeply understanding how this protein is regulated. Posttranslational modifications such as phosphorylation, N-glycosylation, and ubiquitination of PD-L1 have emerged as important regulatory mechanisms that modulate immunosuppression in patients with cancer. In this review, we summarized the latest findings of PD-L1 protein modification and their clinical applications.

## 1. Introduction

Immune checkpoints are the molecules that negatively regulate the activity of T cells. Immune therapy, by targeting checkpoints such as programmed cell death 1 (PD-1) and its ligand programmed death ligand 1 (PD-L1), has shown an important clinical benefit, which has placed tumor immunotherapy in the spotlight [1]. When PD-1 on activated cytotoxic T lymphocytes (CTL) binds to its ligand PD-L1 on the membranes of tumor cells and macrophages in the tumor tissues, immune checkpoint-induced inhibition signals shut down the CTL antitumor immune activity [2]. The antibody of these negative regulators of CTL displays antitumor activity and breaks through historical limitations, leading to durable objective responses in a variety of cancer patients [3, 4]. However, not all patients show persistent remission, and some tumors are completely ineffective in responding to checkpoint blockade. There are no clear demarcating identifiers to distinguish the category of patients who would benefit from the treatment [5]. Therefore, more research studies are now focused on the identification of clinical, histopathological, and genetic biomarkers for anti-PD-1/PD-L1 immunotherapy. Finding of effective biomarkers that could identify patients who would be benefitted is crucial, not only to increase treatment efficacy but also to reduce the risk of those estimated to be unresponsive patients from the side effects of immunotherapy. In addition, identifying these unresponsive patients would be the first milestone to achieve for developing new drugs to overcome immune checkpoint block resistance [6].

**Figure 1:**
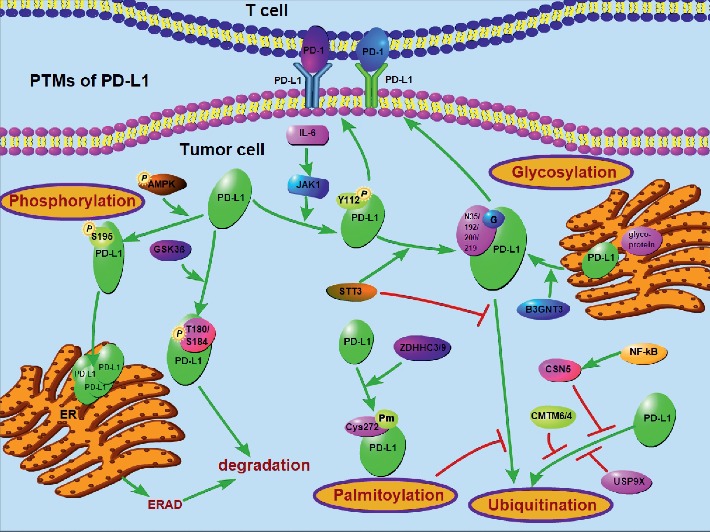
The mechanism of PD-L1 PTMs. Multiple factors are involved in the PTMs of PD-L1 at protein level. The signals implicating the PTMs (N-glycosylation, phosphorylation, polyubiquitination, and palmitoylation) of PD-L1 are presented.

Studies have reported that high tumor mutation burden, immune cell infiltration in tumor tissue, microsatellite instability, and high expression of PD-L1 could be used as predictors for immunotherapy responses [7]. Nonetheless, evidence accumulated in preclinical and clinical studies suggests that the pathological detection of PD-L1 protein levels is neither a consistent nor reliable method in predicting outcomes of anti-PD-L1 treatment [8]. PD-L1 protein levels harbor dynamic changes in the development of the tumor, and corresponding expression changes also occur after immunotherapy, and these dynamic changes are regulated by posttranslational modifications (PTMs) to some extent. PTMs such as glycosylation and phosphorylation affect the structure of the modified protein and its interaction molecule to change its localization and function [9], which suggests that PTM may have a significant effect on the function of PD-L1. Recently, researchers are considering whether PTMs of PD-L1 is a more indicative factor for predicting therapeutic effects of immunotherapy. Since PTMs are commonly used as targets for development of antitumor drugs, the combination of PTM inhibitors may be a new strategy to enhance antitumor immune responses. In the present review, we summarized the latest findings in the most important PTMs of PD-L1 protein, including N-glycosylation, phosphorylation, ubiquitination, and palmitoylation (Figure 1).

## 2. Phosphorylation

Phosphorylation is the most widely studied PTM, and its crosstalk with other PTMs has been significantly proved in recent studies [10]. Metformin activates the AMP-activated protein kinase (AMPK), and then S195 of PD-L1 is directly phosphorylated by p-AMPK, which induces the abnormal glycosylation of PD-L1, leading to its endoplasmic reticulum (ER) accumulation and ER-associated degradation [11]. This process of protein degradation is called endoplasmic reticulum-associated degradation (ERAD) [12]. Adjacent to the glycosylation sites N192, N200, and N219 of PD-L1, there contains the GSK3*β* phosphorylation motif (SxxxTxxxS, where S, serine; T, threonine; and x, any amino acid), GSK3*β* phosphorylates nonglycosylated T180 and S184 of PD-L1 and mediates its degradation [13]. There are many genes encoding proteins with kinase phosphatase activity for PD-L1 [14] such as JAK1. IL-6 activates JAK1, which then phosphorylates Tyr112 of PD-L1; the phosphorylation recruits STT3A to catalyze PD-L1 N-glycosylation and maintains PD-L1 stability by preventing the ubiquitination and degradation of PD-L1 [15]. These research studies revealed that Tyr/Ser/Thr kinases can interact with PD-L1 in the ER region and further regulate phosphorylation and N-glycosylation; these also fully explained how PTMs regulate PD-L1 subcellular localization and thus contributed to the oncogenic functions of intracellular PD-L1 [12, 16].

## 3. Glycosylation

Glycosylation is a very important posttranslational modification which promotes protein folding [17], intracellular transport [18], and functions of immunogenic glycoproteins [19]. Normally, sequential glycosylation is a reaction taking place from the ER to the golgi. First, in the ER region, the precursor glycan Glc_3_Man_9_GlcNAc_2_ bound to Asn-X-Ser/Thr (NXT motif); this motif of glycoprotein is trimmed to Man_8_GlcNAc_2_, and then in the Golgi region, glycan is remodelled [20]. The glycosylated PD-L1 patterns can be detected by western blots (WB), nonglycosylated PD-L1 can be detected at the 33-kDa region, while the majority of PD-L1 is glycosylated and weights from 45 to 55 kDa on WB [13]. The glycosylation manner of PD-L1 is mainly N-glycosylation because the glycosylated PD-L1 was found to be completely decreased by tunicamycin (an N-linked glycosidase inhibitor) rather than O-glycosidase [13, 20, 21]. There are four N-glycosylation sites in the PD-L1 amino acid sequence, namely, N35, N192, N200, and N219 [13]. Glycosylation of coinhibitory molecules is important for regulating the immunosuppressive function and immune surveillance, particularly the glycosylation of PD-L1, which was recently shown to be critical for its function [21, 22].

The effect of glycosylation on PD-L1 is mainly as follows. First, glycosylated PD-L1 is much more stable. The 26S proteasome is the main molecular machine responsible for protein degradation in humans, and nonglycosylated PD-L1 undergoes fast protein degradation by the 26S proteasome [13]. N192, N200, and N219 N-glycosylation prevent PD-L1 from protein degradation [13]. Maher and colleagues found that inhibition of Sigma1 could induce PD-L1 degradation, and Sigma1 was physically associated with glycosyl-PD-L1 [23]. Another study revealed that FKBP51s upregulated PD-L1 expression via catalysing PD-L1 folding, an essential step of glycosylation [24]. As described above, PD-L1 is inserted into the ER and is processed and transported through the secretory pathway. Once glycosylation is dysregulated, the anomalous Man_7-5_GlcNAc_2_ of glycoprotein is identified by endoplasmic reticulum-associated protein degradation (ERAD) and E3 ligase [12]. The abnormal glycosylated protein is polyubiquitinated and transferred from the ER into the cytoplasm, and then this abnormal glycosylated PD-L1 gets degraded by proteasome [25]. Consistent with the previous study [11] which showed that the phosphorylation of PD-L1 was associated with ER glycosylation, Chan et al. reported that PD-L1 phosphorylation recruits STT3A, an ER-associated glycosyltransferase, to catalyze PD-L1 glycosylation and maintain PD-L1 stability [15]. Hsu and colleagues reported that epithelial-mesenchymal transition (EMT) induced N-glycosyltransferase STT3 through *β*-catenin, and subsequent STT3-dependent PD-L1 N-glycosylation stabilizes and upregulates PD-L1 [26]. Li and colleagues revealed that b-1,3-N-acetylglucosaminyl transferase (B3GNT3) stimulates PD-L1 glycosylation, and glycosylated PD-L1 antibody STM10 induces PD-L1 internalization and degradation via lysosomes [21]. All these results revealed that N-linked glycosylation of PD-L1 could make PD-L1 much more stable and lead to a high PD-L1 protein level in cancer. The PD-L1 upregulation results in cancer immune evasion and a failure of checkpoint blockade, and targeting the glycosylation of PD-L1 may be a new therapeutic target of immune therapy.

Second, N-linked glycosylation of PD-L1 is the functional form of PD-L1, which is necessary for PD-L1/PD-1 binding and interaction. Glycosylation is very important in regulating cellular processes such as protein biosynthesis, subcellular localization, stability, and degradation, as it can affect the structure of the protein and its interaction with other molecules [9, 27]. Li and his colleagues found that N-linked glycosylation of PD-L1 by B3GNT3 is required for physical contact between PD-L1 and PD-1, and suppressing N-glycosylation of PD-L1 by tunicamycin (TM), swainsonine (SW), castanospermine (CSP), or 1-deoxymannojirimycin (DMJ) could significantly reduce PD-L1 and PD-1 combination, and coimmunoprecipitation (CO-IP) assay implied that non-glycosylated PD-L1 could not bind to PD-1 [21]. These suggest that developing antibodies targeting glycosylation may help improve the efficacy of immunotherapy.

Third, the removal of N-glycosylation improves PD-L1 clinical detection and predicts anti-PD-1/PD-L1 therapeutic efficacy. PD-L1 immunohistochemistry (IHC) assay is the standard method used in the clinic to stratify patients for immune checkpoint therapy [22]. Lee and colleagues found that the glycosylated PD-L1 has a lower antibody affinity with antibodies against PD-L1, and the deglycosylation of PD-L1 possibly removes the steric hindrance in antibody detection, which can visibly increase the PD-L1 recognition sensitivity by IHC using anti-PD-L1 [22]. The deglycosylation of PD-L1 has the potential to be used as a diagnostic biomarker to predict the response to PD-L1 immunotherapy, and deglycosylation leads to a more accurate assessment of PD-L1 protein level, allowing for a better predictive clinical response to PD-1/PD-L1 immunotherapy [22]. These results suggested that glycosylation is a predictive factor critical for PD-L1 blockade, and removal of it could help us identify patient who would benefit from the immunotherapy.

## 4. Ubiquitination

Ubiquitination is the major way of protein degradation by proteasome, including membrane proteins such as PD-L1 [28], and accumulating evidence demonstrates that proteasomal degradation is the main way of PD-L1 to get degraded [12, 29–32]. These studies suggest that targeting PD-L1 ubiquitination may be an alternative way to improve immune checkpoint therapy. In oral squamous cell cancer (OSCC), ubiquitin-specific peptidase 9, X-linked (USP9X) was found to be upregulated and protected PD-L1 from ubiquitination and degradation, leading to high protein level of PD-L1, resulting in the failure of checkpoint blockade [33]. Abnormal ubiquitination is usually catalyzed by the mutation or ectopic expression of genes that encode E3 ubiquitin ligases or deubiquitinases [34]. *β*-TrCP is a subtype of E3 ligase, PD-L1 contains a *β*-TrCP targeting box (D/LSGXXS), and *β*-TrCP polyubiquitinates nonglycosylated PD-L1 and leads to degradation of PD-L1 in the cytoplasm [13]. Cha and colleagues reported that metformin treatment increased ubiquitination of endogenous PD-L1, ERAD E3 ligase HMG-CoA reductase degradation protein 1 (HRD1) bound to the PD-L1, and promoted its degradation [12].

The ubiquitination of PD-L1 is affected by many factors that participate in tumor initiation. Given that inflammatory molecules often play a major role in modulating immune surveillance [35], TNF-*α* could activate NF-*κ*B, which in result upregulated CSN5. CSN5 is a deubiquitinase that could remove the ubiquitination of PD-L1 and inhibit the degradation of PD-L1 by NF-*κ*B [29]. The protein level of PD-L1 can be regulated during different cell cycles. In multiple human cancer cell lines, Zhang et al. revealed that the fluctuation in the expression level of PD-L1 during different cell cycles was the highest in phase M and early phase G1, followed by a sharp decline in late-phase G1 and phase S, which was mainly because PD-L1 protein expression is regulated by ubiquitination-mediated degradation through cyclin D-CDK4 and the cullin 3-SPOP E3 ligase [32]. Other new molecules were identified as the ubiquitination regulator of PD-L1 such as CMTM6/4. The CMTM6/4 reduces the ubiquitination of PD-L1 by E3 ligase STUB1 and then increases its protein half-life [30]. Since ubiquitination is the major way of regulating protein degradation, we can develop drugs targeting the ubiquitination pathway to improve the ubiquitination of PD-L1 to make PD-L1 more stable, thus reducing the overall level of PD-L1 in tumor cells and improving the effect of immunotherapy.

## 5. Palmitoylation

Palmitoylation is a lipid modification of proteins, and affects the protein functions such as trafficking, activity, stability, and membrane association [36]. Palmitoylation has been found to regulate many cancer-related proteins such as Ras, Wnt, Shh, and epidermal growth factor receptor (EGFR) [36–38]. Typically, palmitoylation mode is a palmitate attached to a cysteine residue via thioester linkage (S-palmitoylation); aspartate-histidine-histidine-cysteine (DHHC) is a palmitoylacyltransferase that catalyzes this process, while acyl-protein thioesterase (APT) mediates depalmitoylation. The palmitoylation stabilizes the PD-L1 in cytoplasmic domain by inhibiting its ubiquitination and then protects PD-L1 from degradation by lysosomes [39]. Yao et al. revealed that palmitoyltransferase ZDHHC3 (DHHC3) is the acetyltransferase that catalyzes PD-L1 in palmitoylation, and inhibiting PD-L1 palmitoylation by 2-bromopalmitate (the palmitoylation inhibitor) or shRNA of DHHC3 could activate anticancer response [39]. Yang et al. revealed that a species conversed palmitoylation site at Cys272 of PD-L1 in the cytosolic domain, and the palmitoylation of PD-L1 by ZDHHC9 maintains protein stability and distribution on the cell surface, resulting in immune escape of the tumor cells [40]. Since the already known palmitoylation of PD-L1 can protect PD-L1 from degradation by lysosomes, further study is needed to verify whether inhibiting ubiquitination is the only way of palmitoylation to make PD-L1 stable and find out the effect of palmitoylation on the detection of PD-L1 in clinical therapy.

## 6. PTMs' Crosstalk of PD-L1

PTMs play an important role in many cellular signaling events. In the case, more than one PTM work interdependently to form a regulation network. The crosstalk makes the regulation of PD-L1 more accurate [10]. While phosphorylation is often laying in the upstream of this crosstalk and could be generated by various predicted kinases, ubiquitination generally leads to protein degradation and lay the downstream of this crosstalk (Figure 1). IL-6-activated JAK1 phosphorylates PD-L1 Tyr112, which recruits STT3A to catalyze PD-L1 glycosylation and maintain PD-L1 stability by preventing its ubiquitination [15]. Likewise, AMPK directly phosphorylates PD-L1 at Ser195 in the ER, and this event causes abnormal glycosylation and polyubiquitination of PD-L1, resulting in PD-L1 protein degradation via ERAD [12]. Findings from these two studies illustrate the typical crosstalk of PTMs in PD-L1, and phosphorylation caused by the external factors and the spatial neighbor of phosphorylation affects glycosylation; then, most of the time, N-glycosylation of PD-L1 prevents PD-L1 from ubiquitination. In a similar way to N-glycosylation, palmitoylation of PD-L1 has been reported to regulate protein stability by blocking ubiquitination of PD-L1 [41], and this lipid modification by DHHC3 in the cytoplasmic domain of PD-L1 stabilizes PD-L1 by blocking its monoubiquitination, consequently suppressing PD-L1 degradation by lysosomes, making palmitoylation and ubiquitination a related process [39].

## 7. Therapeutic Potential of PTMs

Many patients with various types of cancer have significant survival benefits from anti-PD-1/PD-L1 immunotherapy. However, only a subset (10%–40%) of the patients responds to immunotherapy. Resistance to PD-L1 antibodies has greatly reduced the therapeutic effect of immunotherapy on tumors which in turn greatly limits the long-lasting effects and widespread use of immune checkpoint blockades [42, 43]. Therefore, it is crucial to elucidate the underlying mechanism of PD-L1. Numerous studies have shown that PD-L1 is regulated by PTMs, including phosphorylation, glycosylation, palmitoylation, and ubiquitination. The combinations of immune checkpoint blockades with other molecules that regulate PD-1/PD-L1 or interact with PD-1/PD-L1 are being tested in clinical trials, such as cyclin-dependent kinase 4/6 inhibitors plus PD-1/PD-L1 blockade therapies, PARP/c-MET inhibitors plus PD-1/PD-L1 blockade therapies, and EGFR inhibitors plus PD-1/PD-L1 blockade therapies [14, 44]. These studies hint that PTMs may serve as an auxiliary target of PD-1/PD-L1. Since metformin-mediated p-PD-L1 leads to the abnormal glycosylation and degradation of PD-L1, the combination of metformin with immunotherapy anti-CTLA-4 has strong potential to be used in patients with TNBC to improve the immunotherapy effect [12]. JAK1 phosphorylates PD-L1 after IL-6 activation, which is essential for PD-L1's glycosylation by STT3A to protect PD-L1 from ubiquitination and degradation; the combination of IL-6 antibody and Tim-3 (T cell immunoglobulin mucin-3) antibody has been proved to be an effective therapy for liver cancer [15]. A PD-L1 antibody (STM108) that can specifically recognize the B3GNT3-mediated poly-LacNAc moiety on N192 and N200 glycosylation sites of PD-L1 is with potent antitumor activities [21]. 2-deoxyglucose (2-DG) can act as glucose analog to decrease PD-L1 glycosylation, and Shao et al. used it to downregulate PD-L1/PD-1 interaction and decrease PD-L1 translocation and stabilization [45]. The optimization of PD-L1 antibody based on the defucosylation has the potential to enhance the effect of anti-PD-L1 antibodies [46]. To help understand the details, we summarize the latest finding of therapeutic PTMs in Table 1.

Moreover, the PTMs of PD-L1, especially glycosylation, could also be used for improving the clinical detection of PD-L1 predose guidance. IHC method is the general clinical method to detect PD-L1 expression to identify patients who would benefit most from immunotherapy. However, since PD-L1 is often in a heavily glycosylation form, the findings of IHC may be largely misguiding and have a high risk of identifying false-negative patients. Protein deglycosylation of PD-L1 in tumor tissues before IHC assay could make the detection of PD-L1 protein level more accurate, and this allows a more precise prediction of response to PD-L1 immunotherapy [22]. Removal of PD-L1 N-glycosylation with a recombinant glycosidase, peptide-N-glycosidase F (PNGase F) could significantly enhance PD-L1 detection, and assessment of PD-L1 expression after deglycosylation could better predict the effect of anti-PD-L1 antibodies [22].

Most of the therapeutic antibodies approved by FDA mainly target the PD-L1 in the tumor cell surface. The inhibition of PD-L1 palmitoylation not only decreases the level of PD-L1 on the cell membrane but also exhausts the storage of PD-L1 in endosomes [40]. As such, targeting palmitoylation may provide more durable suppression effects for PD-L1 therapy.

## 8. Conclusions

PTMs such as phosphorylation, N-glycosylation, ubiquitination, and palmitoylation of PD-L1 have been approved to be very important for immunotherapy. Given that PTMs are often therapeutic targets for pharmacologic inhibition of cancer, a better understanding of the PTMs of PD-L1 in malignant tumors is of utmost importance. A thorough understanding of the regulatory mechanism of PD-L1 will help us to make a comprehensive evaluation of immune-targeting therapy, further enhance the therapeutic effect, and expand the scope of its use by selecting targeted patients and combination use with other anticancer drugs as well as breaking the limitation of cancer tolerance to immunotherapy. In addition to phosphorylation, glycosylation, ubiquitination, and palmitoylation of PD-L1, there are some other forms of PTMs of PD-L1, such as acetylation and sumoylation, and these may need further investigation to understand how they regulate PD-L1.

There are many questions remaining dim of PTMs study in PD-L1. The supply of ATP is necessary for kinase activation, but the catalysis of ATP uptake into the ER for the phosphorylation of PD-L1 in ER is still relatively and limitedly understood, and how AMPK is located in the ER lumen is also unknown. Most of the therapeutic antibodies approved by the FDA were generally produced by *E. coli* or other host organisms, which do not harbor PTMs. This makes the detection of PD-L1 unsatisfactory, so new technology is warranted for the improvement of the effectiveness of antibody therapy.

## Figures and Tables

**Table 1:** Summary of PTMs of PD-L1.

PTMs	Regulators	PTMs site	Mechanism	Therapeutic strategies	Reference
Phosphorylation	AMPK (metformin-activated)	S195	Abnormal glycosylation of PD-L1 and then PD-L1 ubiquitination and degradation	Metformin + anti-CTLA-4	[12]
	JAK1 (IL-6-activated)	Y112	P-Y112 recruits STT3A to catalyze N-glycosylation, preventing the ubiquitination of PD-L1	IL-6 antibody + Tim-3	[15]
	GSK3*β* (inactivated by EGF/ EGFR, PARP, or c-met)	T180 and S184	Glycosylations of N192/200/219 antagonize PD-L1 and GSK3*β* interaction, and the P-T180/S184 triggers interaction with E3 ligase *β*-TrCP	PRAP or c-met inhibitor	[14, 44]

Glycosylation	Sigma1	Cochaperones	Facilitates glycosylated PD-L1	IPAG	[23]
	FKBP51s	Cochaperones	Facilitates glycosylated PD-L1	SAFit	[24]
	B3GNT3	N192 and N200	Stimulates PD-L1 glycosylation, and prevents PD-L1 from internalization and degradation via lysosomes	gPD-L1 antibody-drug conjugate	[21]
	STT3	N35, N192, N200, and N219	Stimulates PD-L1 glycosylation, and prevents PD-L1 from internalization and degradation via lysosomes	Etoposide + Tim-3	[26]

Ubiquitination	*β*-TrCP	D/LSGXXS	Polyubiquitinate nonglycosylated PD-L1 leading to degradation of PD-L1	Resveratrol	[13]
	E3 ligase HRD1		Recognizes abnormal glycosylation of PD-L1, and promotes its degradation in ERAD	Metformin + anti-CTLA-4	[12]
	E3 ligase SPOP (E3 ligase CDK4)		Degradation of PD-L1	CDK4/6 inhibitor palbociclib or ribociclib	[32]
	E3 ligase STUB1 (upregulated by CMTM6/4)		Degradation of PD-L1	CMTM6 depletion	[30, 31]
	CSN5 (activated by TNF-*α* and NF-*κ*B)		Removes ubiquitination of PD-L1 and inhibits degradation	Curcumin + anti-CTLA-4	[29]
	USP9X		Protects PD-L1 from ubiquitination and degradation		[33]

Palmitoylation	DHHC3/9	Cys272	Inhibits ubiquitination, and protects PD-L1 from degradation by lysosomes	2-bromopalmitate	[39]
